# The risk of organ-based comorbidities in psoriasis: a systematic review and meta-analysis^☆^

**DOI:** 10.1016/j.abd.2021.10.007

**Published:** 2022-07-15

**Authors:** Xuemei Tang, Ling Chen

**Affiliations:** a Southwest Medical University, Luzhou, Sichuan, China; b Department of Dermatology, Daping Hospital, Army Medical University, Chongqing, China

**Keywords:** Comorbidity, Meta-analysis, Psoriasis, Risk, Systematic review

## Abstract

**Background:**

The close relationship between psoriasis and concomitant diseases is widely accepted. However, a comprehensive analysis of organ-based comorbidities in psoriasis is still lacking.

**Objective:**

The authors aimed to present the risk of organ-based comorbidities in psoriasis by comparing the general population.

**Methods:**

The authors retrieved a search of Pubmed, EMBASE, and Cochrane databases for studies reporting organ-based comorbidities in psoriasis versus the general population. Observational studies that met the following criteria were assessed: 1) Psoriasis diagnosis; 2) Cardiovascular or kidney or liver or respiratory or cerebrovascular outcomes; 3) Comparison group of individuals without psoriasis. Pooled Relative Risks (pRRs) and 95% Confidence Intervals (CIs) were calculated by using the random-effect model.

**Results:**

Fifteen observational studies with 216,348 psoriatic patients and 9,896,962 individuals from the general population were included. Psoriasis showed a greater risk of organ-based comorbidities. Compared to the general population, pRR for all organ-based comorbidities was 1.20 (95% CI 1.11‒1.31) in psoriasis, and pRR was lower in mild 0.61 (95% CI 0.46‒0.81) than in moderate/severe patients. pRR was 1.20 (95% CI 1.11‒1.30) for cardiovascular, 1.56 (95% CI 1.20‒2.04), and 1.75 (95% CI 1.33‒2.29) for cerebrovascular and liver diseases, respectively. pRR for coexisting renal and cardiovascular events was 1.09 (95% CI 1.01‒1.18). pRR for coexisting renal and cerebrovascular events was 1.28 (95% CI 0.99‒1.66). pRR for coexisting renal and liver diseases was 1.46 (95% CI 1.10‒1.94). pRR for coexisting cardiovascular and liver diseases was 1.41 (95% CI 1.11‒1.80).

**Study limitations:**

There is heterogeneity.

**Conclusion:**

Psoriasis has a higher risk of single and multiple organ-based comorbidities than the general population. The present study will further improve attention to psoriasis as a systemic inflammatory disease.

## Introduction

Psoriasis is a chronic inflammatory disease, affecting approximately 2%‒3% of the global population.^1^, ^2^, ^3^, ^4^, ^5^, ^6^, ^7^, ^8^ The pathogenesis of psoriasis is believed to be the result of the interaction of genetic, environmental, and immune factors.^9^, ^10^, ^11^ Psoriasis has been considered to be a systemic disease that may increase risks of cardiovascular disease, metabolic syndrome, and other comorbidities.^12^, ^13^, ^14^ The comorbidity mechanism of psoriasis may be related to the release of pro-inflammatory molecules during chronic inflammation.^3^, ^15^, ^16^, ^17^ More and more evidences suggest that severe or relapsing psoriasis tends to be a systemic inflammation disease.^18^, ^19^ Studies have shown that 13% of psoriatic patients are associated with ischaemic heart disease, 12% associated with diabetes mellitus, and 36% associated with arterial hypertension.^20^ There are meta-analyses about psoriasis with single diseases, e.g. Chronic Obstructive Pulmonary Disease (COPD)^21^ and diabetes.^22^ However, there is no comorbidity data at levels of organs.

Although the close relationship between psoriasis and concomitant diseases has been commonly accepted, and the risk of a single specific disease in psoriasis has been intensively studied.^21^, ^23^, ^24^ However, a comprehensive analysis of comorbidities in terms of organs in psoriasis is still lacking. In this study, the authors aimed to investigate the differences between psoriasis and the general population in terms of different organ-based comorbidities. The analysis was not limited to purely merger values and tried to find association patterns between them.

## Material and methods

### Literature review and search strategy

This meta-analysis was registered in PROSPERO (CRD 42020211821), following the PRISMA 2020 checklist.^25^ The authors searched Pubmed, EMBASE, and Cochrane Library databases, including all studies from the respective inception of these databases to October 14, 2020. The investigator performed the literature retrieval, research selection, data extraction, and quality assessment.

The search strategy included a variety of terms related to psoriasis and organ-based comorbidities. The detailed search strategies of Pubmed, Embase, and Cochrane Library were put in the supplementary material.

### Selection of studies

According to the PECO strategy, articles that met the following criteria would be included. Population: the authors included studies that contained both psoriasis and the general population; Exposure: psoriasis with regular diagnosis (no age limit); Comparative: the people without psoriasis and from the same research environment as psoriasis; Outcome: the record results were organ-based comorbidities (cardiovascular diseases, kidney diseases, liver diseases, respiratory diseases, spleen diseases, and cerebrovascular system-related diseases). Publications with insufficient and reduplicated data were excluded. The restriction of study type and language were not considered in the initial search.

### Data extraction and assessment of study quality

Full text of articles meeting the eligibility criteria was selected for data extraction. Data was extracted by the first author, year of publication, country, demographic, number of patients, and follow-up time. For example, the authors intended to include studies that were clearly defined as diseases with the kidney, such as chronic kidney disease, etc. In addition, studies that had nothing, but abnormal testing data were excluded. If a study provided data with different types of psoriasis (mild, moderate, and severe), the authors combined them to obtain an estimate of all psoriatic patients. In order to ensure the accuracy of data extraction, any difference found in the data can be addressed by referring to the original article. The Newcastle-Ottawa Quality Assessment Scale was used to assess the quality of included studies, with a maximum score of 9 points, representing the highest quality. The scale assessed the quality of each study in three areas, including (1) Case and control subjects’ recruitment; (2) Comparability between the two groups; (3) Identification of results of interest. The characteristics of the studies and evaluation results were displayed in Table 1.

**Table 1:** Characteristics of included studies: psoriasis and comorbidities.

Study	Region	Study period	Data sources	Mean age, (y); Female (%)		N° of psoriatic patients		N° of Control		Specific comorbidities	NOS
				Psoriasis	Control	All	Comorbidities	All	Comorbidities		
Ahlehoff O, 2011^30^	Danish	2002–2006	The Danish National Patient Register	69.5 (12.1); 36.58	70.6 (13.5); 38.72	462	199	48935	20356	CVD, KD, PD	6
Ahlehoff O, 2012^31^	Danish	1997.01–2006.12	The Danish National Patient Register	46.1 ± 16.9; 49.72	43.7 ± 19.7; 49.03	39558	530	4478926	55660	CVD, KD, CD, PD	7
Armstrong AW, 2013^32^	US	2004–2009	University of California Davis	53 (40‒63); 59.19	53 (40‒63); 51.11	2078	1776	6234	3616	CVD, CD, PD	6
Charlton R, 2018^33^	UK	1998.01–2014.12	The UK Clinical Practice Research Datalink	49 (39‒59); 50.95	49 (39‒59); 50.95	27132	8444	27132	7051	CVD, CD	7
Chiang CH, 2011^34^	Taiwan	Before 2007	Longitudinal Health Insurance Database 2007	45.3 ± 17.4; 43.69	43.5 ± 17.4; 22.14	2783	1066	13910	5145	CVD, KD	6
Feldman SR, 2015^35^	US	2007.01–2012.03	The OptumHealth Reporting and Insights claims database	47.62; 44.50	47.62; 44.50	5492	881	5492	499	KD, CD, PD, LD	7
Khalid U, 2015^36^	Danish	1997.01–2011.12	The Danish National Patient Register	43.3 (15.8); 51.48	41.9 (19.9); 5 0.71	70665	1292	5036959	105321	CVD, KD, PD	7
aLee S, 2017^37^	US	2010.11–2015.10	The US Department of Defense population	48.1; 51.84	48.1; 51.84	7249	2932	72490	22367	CVD, CD	7
Min C, 2019^26^	Korean	2002‒2013	The Korean National Health Insurance Service-National Sample Cohort	NR; 43.32	NR; 43.32	11071	5187	44284	20298	CVD, CD	7
Prasada S, 2020^29^	US	2000.01–2019.01	The Northwestern Medicine Enterprise Data Warehouse	49.72 (15.95); 51.71	48.60 (16.65); 57.34	5365	1536	19358	5241	CVD, KD	7
Schell C, 2015^38^	German	NR	Eberhard Karls University Tübingen	48.3 ± 15.6; 43.33	49.1 ± 18.6; 60.00	300	158	300	150	CVD, KD, PD, LD	6
Tollefson MM, 2018^28^	US	2004.01–2013.12	Optum Laboratories Data Warehouse	12.0 (4.4); 53.52	12.0 (4.4); 53.52	29957	1200	29957	646	CVD, LD	8
Vena GA, 2010^39^	Italy	2001‒2005	The Health Search/Thales Database	52.5 (16.5); 47.41	52.4 (16.4); 52.59	3516	399	17580	1425	CVD, PD	6
Yang YW, 2011^27^	Taiwan	2006.01–2007.12	Longitudinal Health Insurance Database2000	NR, >18; 32.58	NR, >18; 67.42	1685	1471	5055	3651	CVD, KD, CD, PD, LD	8
Yeung H, 2013^40^	UK	NR	The Health Improvement Network	46 (37‒55); 49.43	46 (36‒55); 52.91	9035	1945	90350	17502	CVD, KD, CD, PD, LD	6

NR, Not Reported; NOS, Newcastle–Ottawa Quality Assessment Scale; CVD, Cardiovascular Disease; CD, Cerebrovascular Disease; KD, Kidney Disease; PD, Pulmonary Disease; LD, Liver Disease; US, United States; UK, United Kingdom.

a The data of study included Psoriatic arthritis.^37^

### Statistical analysis

Statistical analyses were performed by RevMan 5.4 (the Cochrane collaboration) and Stata version 12.0 (StataCorp LP, College Station, TX77845). The results were reflected in pooled RR (pRR) and 95% CI. All statistical tests were bilateral. The p-values of less than 0.05 were considered significant. The Inconsistency test (I²) statistic was used to assess the heterogeneity. The I² value of 0%, 25%, 50%, and 75% indicated no, low, medium, and high heterogeneity, respectively. Sensitivity analyses, subgroup analyses, and meta-regression were performed to identify potential sources of heterogeneity. Besides, the funnel plots and Egger’s weighted regression were used to assess potential publication bias.

The pRRs and corresponding 95% CIs were estimated by a random-effect meta-analysis. A logarithmic transformation was performed for the estimated RRs of maximum adjusted effect size and the CIs. pRRs were subjected to Z-test and sensitivity analysis and used to assess the combined stability effect. Publication bias was measured by constructing funnel plots. A symmetrical inverted funnel indicated that there is no high likelihood of publication bias. Finally, a study was removed to assess its impact on the merger. The similarity of combined RRs before and after removing the study indicated that there was a high certainty in the results. When heterogeneity existed between studies, the authors applied the random-effect model and used the Mantel-Haenszel analysis method.

## Results

### Study selection

**Figure 1:**
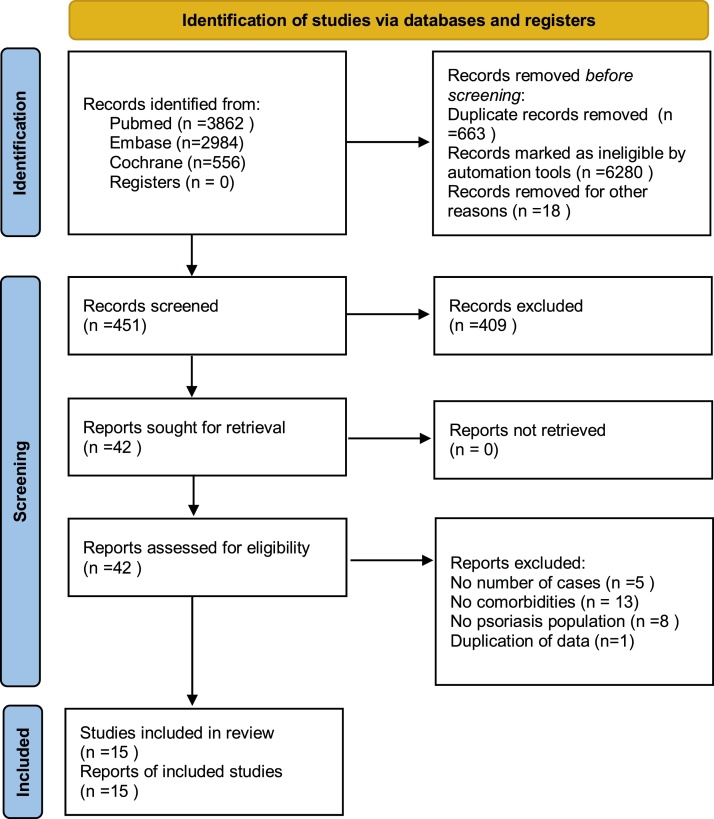
Flow diagram of study selection. PRISMA 2020 flow diagram for new systematic reviews which included searches of databases and registers only.

The search strategy (Fig. 1) produced 7,412 potentially relevant references (3,862 in Pubmed, 2,984 in EMBASE, and 566 in the Cochrane Library). Finally, fifteen retrospective cohort studies remained, containing 216,348 psoriatic patients and 9,896,962 general people. All studies were based on a managed database, relied on diagnostic codes to identify and verify the diagnosis of psoriasis and organ-based comorbidities.

### Study characteristics

The main clinical characteristics and quality evaluation of the included studies were summarized in Table 1. These studies were conducted in seven countries: the United States, the United Kingdom, Germany, Denmark, Italy, Taiwan, and South Korea. The mean age of psoriatic patients ranged from 12.0 to 69.5 years, and females accounted for 32.58% to 59.19%. For the general population, the mean age ranged from 12.0 to 70.6 years, and females accounted from 22.14% to 67.42%. Only two studies did not report the average age,^26^, ^27^ and one study mainly investigated children with psoriasis.^28^ So, the span of age characteristics was large after synthesizing data. There was little specific organ-based comorbidities data of race. Therefore, no subgroup analysis was conducted on race.^28^, ^29^

### Psoriasis and all organ-based comorbidities

**Figure 2:**
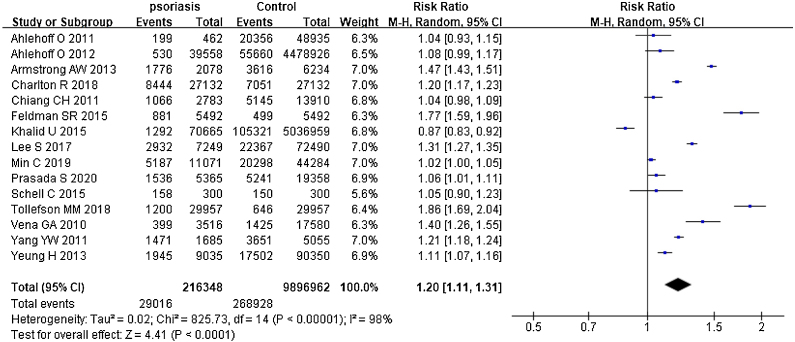
Forest plot of the risks of comorbidities in psoriatic patients comparing the general population.

The risks all included organ-based comorbidities between psoriatic patients and the general population were described in Fig. 2. The authors mainly studied the involvements of the heart, kidneys, lung, brain, and liver. In 15 studies,^26^, ^27^, ^28^, ^29^, ^30^, ^31^, ^32^, ^33^, ^34^, ^35^, ^36^, ^37^, ^38^, ^39^, ^40^ they contained 216,348 psoriatic patients and 9,896,962 control subjects. It showed that psoriatic patients had a significantly increased risk of these organ-based comorbidities, and pRR was 1.20 (95% CI 1.11‒1.31, I² = 98%, p < 0.001). After removing the study involving mainly children,^28^ pRR became to 1.17 (95% CI 1.08‒1.27, I² = 98%, p < 0.001).

### Psoriasis and specific organ-based comorbidities

**Figure 3:**
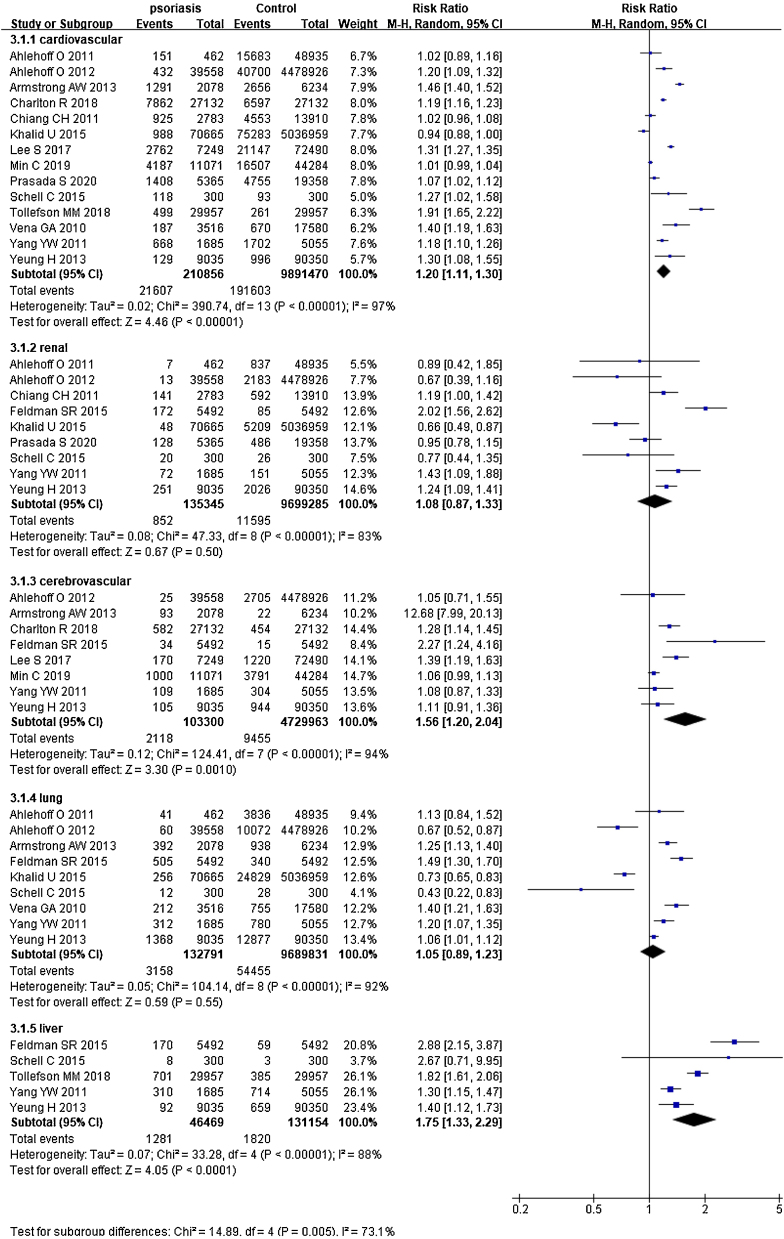
Forest plot of specific comorbidity risk in psoriatic patients compared with the general population (cardiovascular, renal, cerebrovascular, lung, liver diseases).

In 14 studies,^26^, ^27^, ^28^, ^29^, ^30^, ^31^, ^32^, ^33^, ^34^, ^36^, ^37^, ^38^, ^39^, ^40^ summing 210,856 psoriatic patients and 9,891,470 controls. The pRR for cardiovascular events was 1.20 (95% CI 1.11‒1.30, I² = 97%, p < 0.001), significantly increased comparing the general population. In 9 studies^27^, ^29^, ^30^, ^31^, ^34^, ^35^, ^36^, ^38^, ^40^ involved 135,345 psoriatic patients and 9,699,285 control subjects, pRR for renal diseases was 1.08 (95% CI 0.87‒1.33, I² = 83%, p < 0.001). In 8 studies^26^, ^27^, ^31^, ^32^, ^33^, ^35^, ^37^, ^40^ with 103,300 psoriatic patients and 4,729,963 control subjects, pRR of cerebrovascular disease events was 1.56 (95% CI 1.20‒2.04, I² = 94%, p < 0.001). In 9 studies^27^, ^30^, ^31^, ^32^, ^35^, ^36^, ^38^^,^^40^ with 132,791 psoriatic patients and 9,689,831 control subjects, pRR for the occurrence of pulmonary disease events was 1.05 (95% CI 0.89‒1.23, I² = 92%, p < 0.001). In 5 studies^27^, ^28^, ^35^, ^38^^,^^40^ involved 46,469 psoriatic patients and 131,154 control subjects, pRR for the occurrence of liver diseases was 1.75 (95% CI 1.33‒2.29, I² = 88%, p < 0.001), and it was remarkably higher than general population (Fig. 3).

### Psoriasis and multiple organ-based comorbidities

**Figure 4:**
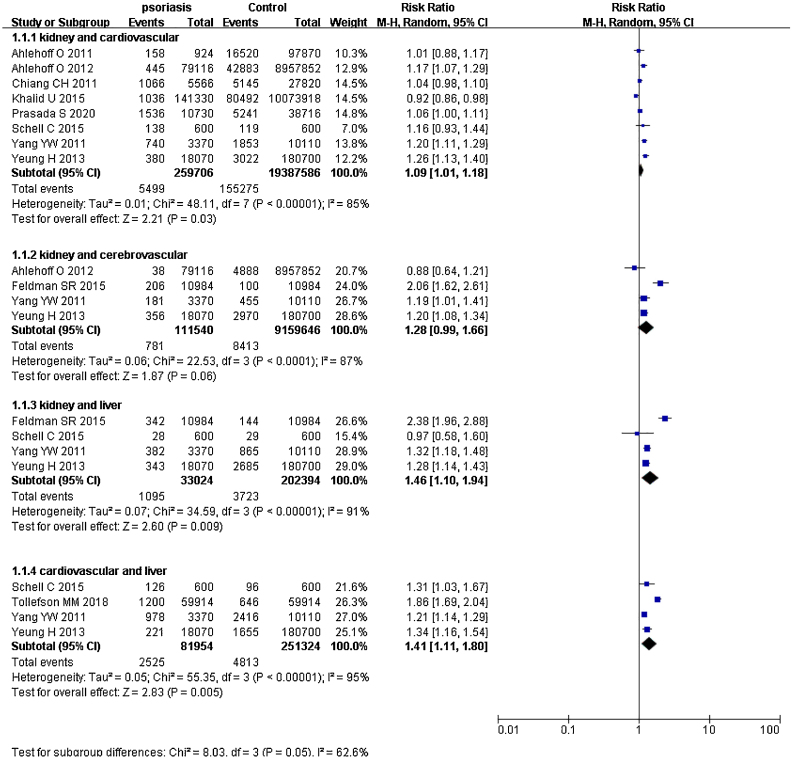
Forest plot of coexisting comorbidity risk in psoriatic patients compared with general population.

Psoriatic patients had been reported to suffer from two or more comorbidities simultaneously.^41^, ^42^, ^43^, ^44^, ^45^, ^46^ Hence, the authors extracted data of two organs from included studies and calculated pRRs (Fig. 4). pRR for renal diseases *plus* cardiovascular events was 1.09 (95% CI 1.01‒1.18, I² = 85%, p < 0.001). pRR for renal diseases *plus* cerebrovascular events was 1.28 (95% CI 0.99‒1.66, I² = 87%, p < 0.001). pRR for renal diseases *plus* liver diseases were 1.46 (95% CI 1.10‒1.94, I² = 91%, p < 0.001). pRR for cardiovascular events *plus* liver diseases were 1.41 (95% CI 1.11‒1.80, I² = 95%, p < 0.001).

### Organ-based comorbidity risks in mild and moderate/severe psoriasis

Three studies were subdividing psoriatic patients into mild and moderate/severe subgroups.^31^, ^32^, ^36^ They included 97,285 patients with mild psoriasis and 15,016 patients with moderate/severe psoriasis in total. pRR of organ-based comorbidity risk was 0.61 (95% CI 0.46‒0.81, I² = 94%, p < 0.001) for mild psoriasis, which was lower than that of moderate/severe psoriatic patients (Supplementary Fig. S1).

### Sensitivity analyses

Sensitivity is an important indicator of literature quality and heterogeneity. One study was excluded to estimate the effect of pRRs at a time. There was no obvious change and a significant decrease in heterogeneity. Sensitivity analyses were performed to assess which study provided greater heterogeneity in the data by the Galbraith plot. All studies showed stable and reliable results (Supplementary Fig. S2). After careful evaluation, it was concluded that every study was not sensitive to pRRs and could not be arbitrarily excluded. As the score results were assessed by Newcastle-Ottawa Quality Assessment Scale for all included studies in Table 1. All studies scored at least 6 points, which was in line with the requirements for continuing study. Meanwhile, Stata's Metaninf command was used to assess the impact of a single study. After excluding one study at a time, the point of estimate did not fall out of 95% CI (Supplementary Fig. S3).

### Publication bias

The funnel plot was utilized to analyze the publication bias of 15 studies. The outcome of the graph showed a certain degree of publication bias. The authors further used the Egger method to check and quantify the funnel plot. The p-value was checked to determine whether there was publication bias. The T-value in bias (p = 0.854) meant that there was no publication bias (Supplementary Fig. S4).

### Study quality

Regarding the quality of all studies, the NOS score ranged from 6 to 8. The authors determined that 6 studies had a low risk of bias, 7 studies had a medium risk of bias, and 2 studies had a high risk of bias (Table 1).

### Subgroup analysis

The authors conducted the subgroup analyses, including regions (Western vs. Asian countries), study period (covering 2000 vs. after 2000), and funding (with vs. without). The research region of the three studies was Asia,^26^, ^27^, ^34^ the rest was the West. The outcomes showed that Asian countries with pRR of 1.09 (95% CI 0.95‒1.24, I² = 98%, p < 0.001) and 1.24 (95% CI 1.12‒1.37, I² = 98%, p < 0.001) for Western countries. The I² was 55.5% and p-value was 0.13 between the subgroups (Supplementary Fig. S5).

Two studies had not clearly pointed out the study duration,^38^, ^40^ and the rest studies were divided into two groups for further discussion. Four researches’ periods were over the year of 2000,^31^, ^33^, ^34^, ^36^ and pRR was 1.04 (95% CI 0.89‒1.21, I² = 97%, p < 0.001). pRR of the rest researches (after 2000) was 1.31 (95% CI 1.17‒1.47, I² = 99%, p < 0.001). The I² was 82.7% and the p-value was 0.02 between the subgroups (Supplementary Fig. S6).

There was some research that had no funding,^27^, ^32^ while the rest of the studies were funded. The analysis showed that pRR of unfunded research was 1.33 (95% CI 1.10‒1.62, I² = 99%, p < 0.001), and pRR of funded ones was 1.18 (95% CI 1.09‒1.29, I² = 98%, p < 0.001), The I² was 18.7% and p-value was 0.27 between the subgroups (Supplementary Fig. S7).

These results of subgroup analyses, including regions (Western vs. Asian countries), study period (covering 2000 vs. after 2000), and funding (with vs. without), had not provided any specific source of heterogeneity.

### Meta-regression

According to the characteristics of included studies, new concomitant variables were generated after dummy variables were assigned according to regions (Western vs. Asian countries), study period (covering 2000 vs. after 2000), and funding (with vs. without). The logarithmic LogRR of each study effect indicator RR was the dependent variable. The meta regressions were all carried out (p > 0.05), but there was no source of heterogeneity.

## Discussion

The present study is the first meta-analysis of comorbidity risk in terms of organs in psoriatic patients. These risks of organ-based comorbidity were consistent with the previously published data at single-disease level. For example, previous studies showed that psoriatic patients had a higher risk of hypertension (OR = 1.58, 95% CI 1.42‒1.76),^47^ end-stage renal disease (RR = 1.29, 95% CI 1.05‒1.60),^48^ stroke (OR = 1.08, 95% CI 1.00‒1.16),^49^ non-alcoholic fatty liver disease (OR = 2.15, 95% CI 1.57‒2.94),^24^ and COPD (OR = 1.90, 95% CI 1.36‒2.65).^21^ The authors found that psoriatic patients had a significantly higher risk of organ-based comorbidities than the general population. For example, comorbidity pRR was 1.20 for the overall cardiovascular organ, 1.75 for the whole liver, 1.56 for the whole cerebrovascular organ, 1.08 and 1.05 for the entire kidney organ and lung organs, respectively. In the cardiovascular, liver, and cerebrovascular, organ-based comorbidity risks of psoriasis were remarkably higher than in the general population. Although there was no significant difference between psoriasis and controls in relation to renal and pulmonary diseases, the results can also provide some reference for future research. In addition, compared with mild psoriatic patients, the authors demonstrated that moderate/severe psoriatic patients had a more notably increased risk of organ-based comorbidities.

Previous studies had shown that psoriatic patients may suffer from two or more comorbidities simultaneously, e.g., heart failure combined with chronic kidney disease,^43^ gout or acute kidney injury combined with hypertension, liver cirrhosis, or cerebrovascular disease.^44^, ^45^, ^46^ To find out these combined comorbidity risks in psoriasis, the authors pooled data from multiple organs. The authors found that pRR for renal diseases *plus* cardiovascular events in the same psoriatic patient was 1.09 (95% CI 1.01‒1.18), 1.28 (95% CI 0.99‒1.66) for renal diseases *plus* cerebrovascular events, 1.46 (95% CI 1.10‒1.94) for renal diseases *plus* liver diseases, and 1.41 (95% CI 1.11‒1.80) for cardiovascular events *plus* liver diseases. These values showed that psoriatic patients had significantly higher risks of multiple organ-based comorbidities, not only single organ-based comorbidities.

In order to further analyze the relationship between organ-based comorbidity risks of psoriasis and study year and patients’ region, the authors compared several sets of data. Due to the diverse characteristics of the years included in the studies, the authors took the year 2006 as the demarcation point, based on the principle of pursuing maximum similarity. Six groups of meaningful data were selected,^27^, ^30^, ^31^, ^32^, ^35^, ^37^ and pRR was 0.23 (95% CI 0.05‒1.00, I² = 100%, p < 0.001) after merging data (Supplementary Fig. S8). This suggested that psoriatic patients after the year of 2006 were more likely to suffer from organ-based comorbidities. As few studies were included in the East, three groups matched with the characteristics of studies in East were used for comparative study.^26^, ^27^, ^32^, ^33^, ^34^, ^39^ After merging data, pRR was 1.73 (95% CI 1.07‒2.77, I² = 100%, p < 0.001) (Supplementary Fig. S9). The result suggested that Asian psoriatic patients were more likely to suffer from organ-based comorbidities. These conclusions need more data for further confirmation.

The pathogenesis of psoriasis and organ-based comorbidities may be mediated by certain representative cytokines, e.g., Interleukin-17 (IL-17),^50^, ^51^, ^52^, ^53^ Interleukin-23 (IL-23),^54^ and Tumour Necrosis Factor-ɑ (TNF-ɑ).^55^, ^56^ They can reach the body anywhere through the blood circulation and may mediate systemic inflammatory responses in psoriasis.^57^, ^58^, ^59^, ^60^, ^61^ Whether the inflammatory cytokines act on each system together or singly? Whether the effects are generated by the same molecular mechanism and immune-inflammatory pathway? These questions are unclear and still need further study. Actually, the cytokines behind the pathogenesis of each organ may be the same or absolutely different. The existing research has shown that main inflammation cytokine behind psoriasis with joints or diabetes is TNF-ɑ.^62^, ^63^, ^64^, ^65^, ^66^ However, the cytokine in psoriasis involving the cardiovascular system may be IL-17 or IL-23.^67^, ^68^, ^69^ All these still need further investigation.

The present study can provide a reference for effective clinical treatments. Lately, biological antibody therapy plays a promising role in the control of psoriatic morbidity and mortality.^70^, ^71^, ^72^, ^73^, ^74^, ^75^ If molecular mechanisms and dysregulated target cytokines in involved organs are different, then the choice of biological antibody should be totally different.^76^, ^77^, ^78^, ^79^, ^80^ No biological antibody is a panacea,^81^, ^82^, ^83^, ^84^ and the authors should try to provide patients with more individualized treatment plan according to organ-based comorbidities that psoriasis involved.

There are several limitations in the meta-analysis. First, there is heterogeneity. It was considered that heterogeneity was normally distributed and acceptable. The statistical heterogeneity did not affect the outcome. It was observed that the rate of heterogeneity in meta-analysis of general prevalence was always high. It was related to a huge number of study objects and the nature of observational studies. In this study, subgroup analysis and meta-regression were conducted to try to find the source of heterogeneity. The authors also used a random-effects model to address potential heterogeneity. However, each study was qualitatively similar to the analysis of all organ-based comorbidities and individual organ-based comorbidities. Secondly, some systematic analyses, meta-regression, and subgroup analyses lacked high statistical power because of the small number of included studies. Care should be taken when analyzing these data. Thirdly, the topic of the present study is novel, and there is no study on organ-based comorbidities of psoriasis. For the PECO approach, it is different from the traditional PECO expression and may cause confusion. The study population included the exposure group plus the control group, i.e., the Population contains Exposure and Comparative. However, the present study’s results can still provide a reference for clinicians in the management of psoriasis comorbidities.

## Conclusions

The authors show that psoriatic patients have increased risks of both single and multiple organ-based comorbidities. None of these has been reported in published literature. These results suggest that systemic inflammation control should be observed in the treatment, especially for moderate/severe patients.

## Financial support

This study was supported in part by the 10.13039/501100001809National Natural Science Foundation of China (nº 81771783, 82073444).

## Authors’ contributions

Xuemei Tang: Approval of the final version of the manuscript; critical literature review; data collection, analysis, and interpretation; effective participation in research orientation; intellectual participation in propaedeutic and/or therapeutic; management of studied cases; manuscript critical review; preparation and writing of the manuscript; statistical analysis; study conception and planning.

## Conflicts of interest

None declared.
