# Supplementary material for: The risk of organ-based comorbidities in psoriasis: a systematic review and meta-analysis
Source: An Bras Dermatol. 2022 Jul 15;97(5):612–23. doi: 10.1016/j.abd.2021.10.007 (PMC9453528; doi:10.1016/j.abd.2021.10.007)
Supplement: Supplementary file 1 [file mmc1.docx]

**ABD-D-21-00377_ Supplementary material**

**Supplementary Figure 1** Forest plot of comorbidity risk in mild and moderate/severe psoriatic patients.


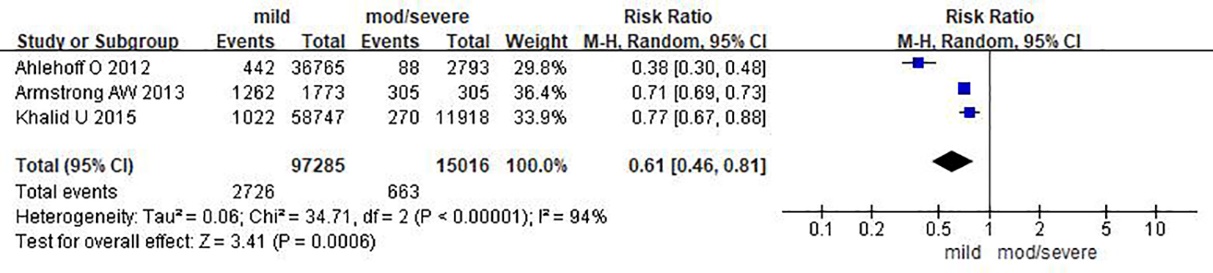


**Supplementary Figure 2** Galbraith diagram was plotted by using the GALbr command.

**
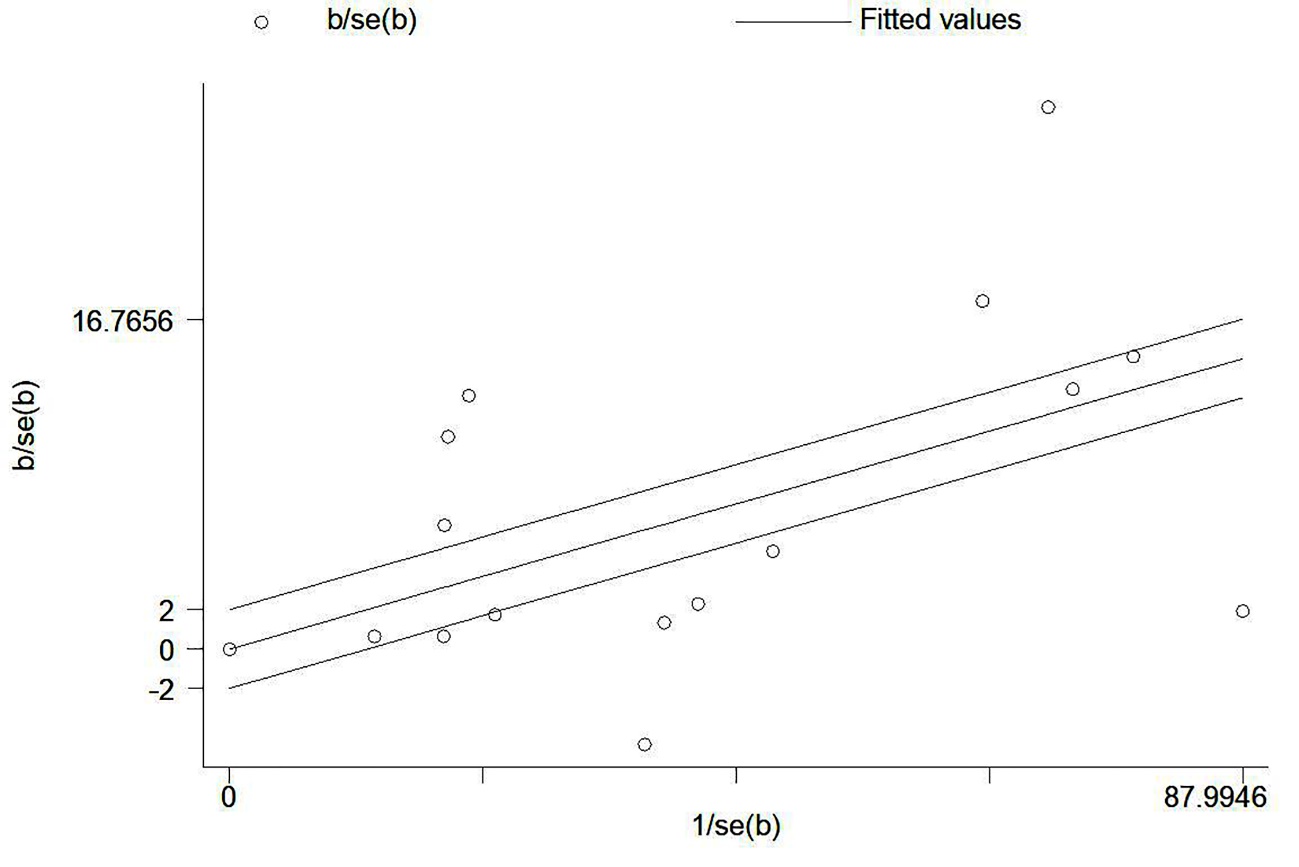
**

**Supplementary Figure 3** Stata's Metaninf command was utilized to investigate the effect of a single study on the pooled effect size.


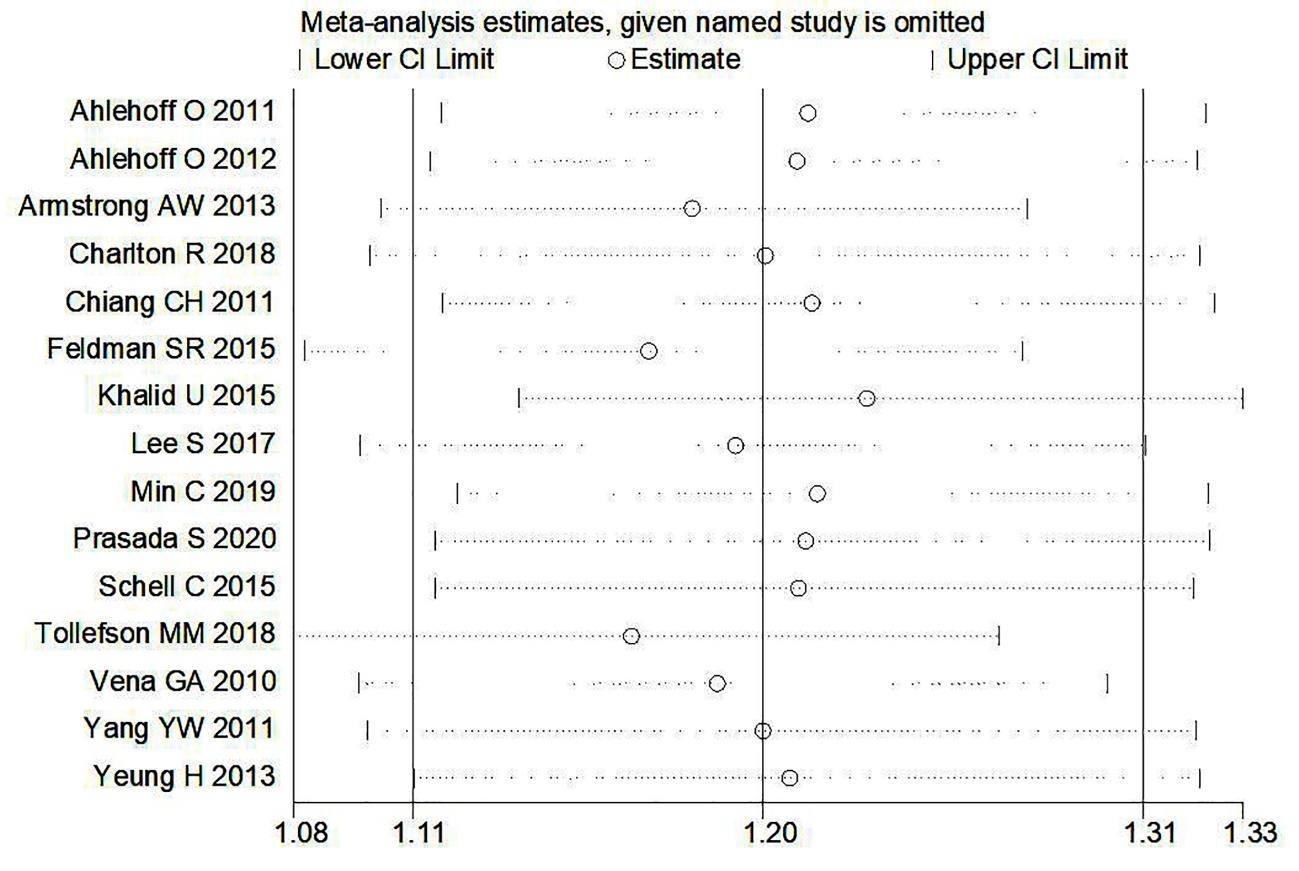


**Supplementary Figure 4** Utilizing the Metabias command and the Egger method to show abias test.


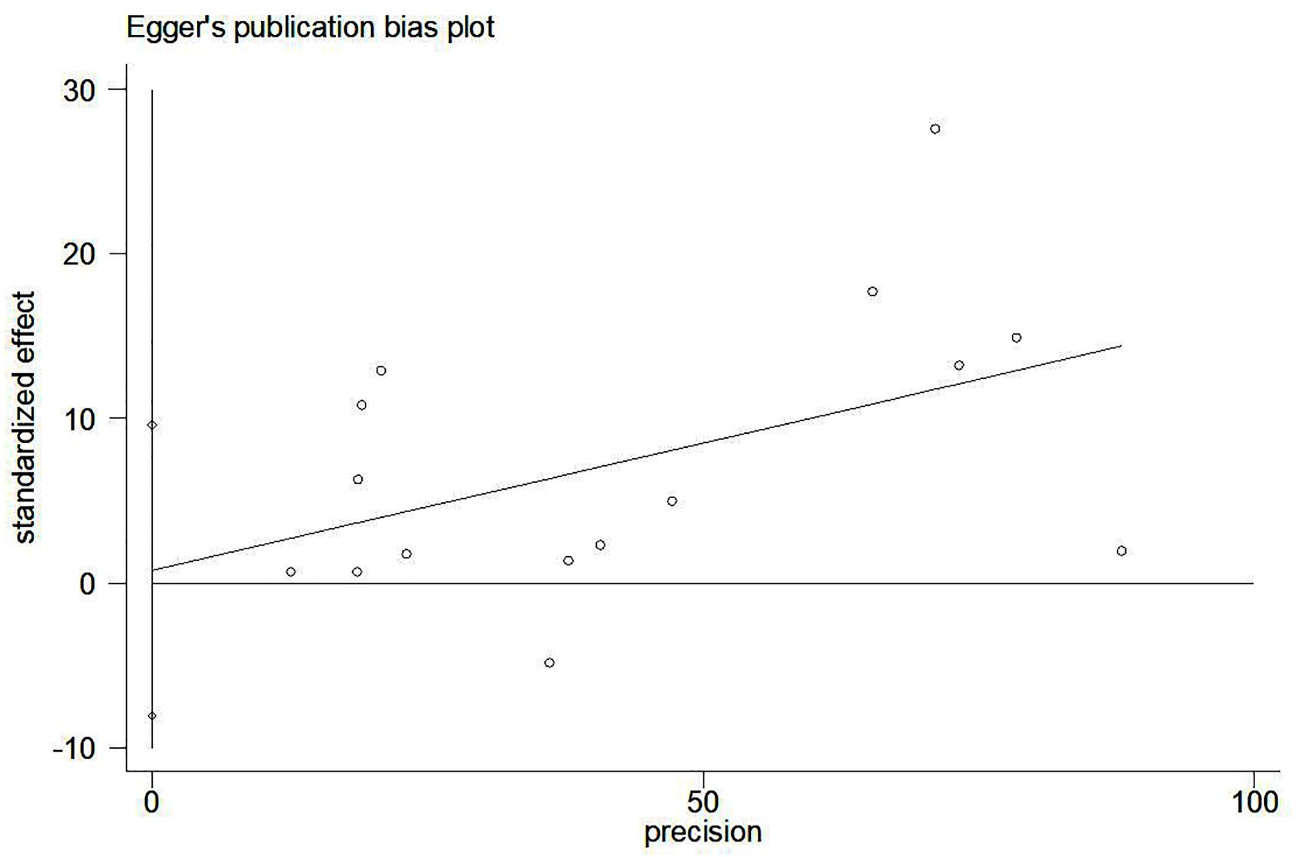


**Supplementary Figure 5** Subgroup analyses in Western countries vs. Asian countries.


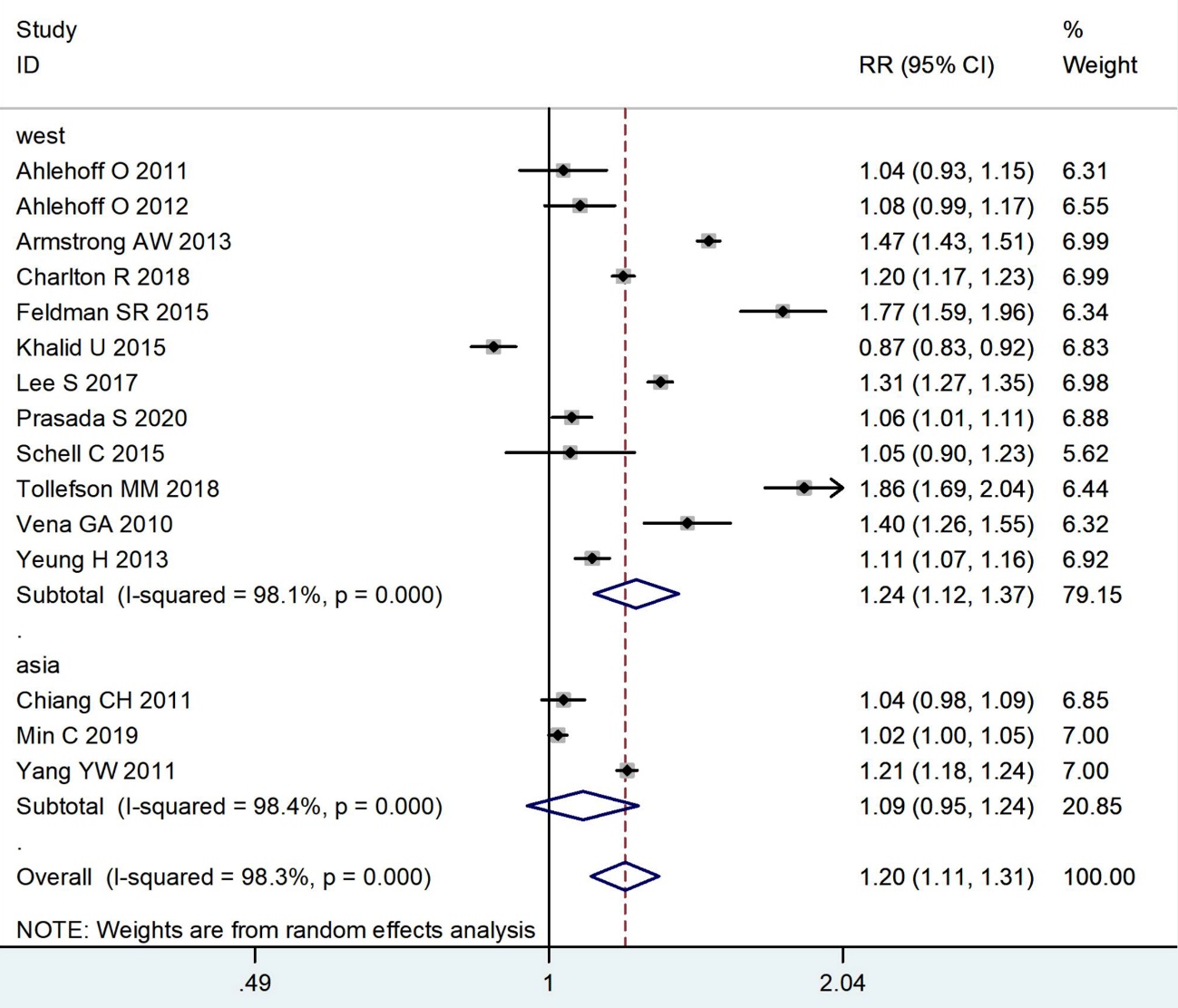


**Supplementary Figure 6** Subgroup analyses in data of comorbidities covering and after the year of 2000.


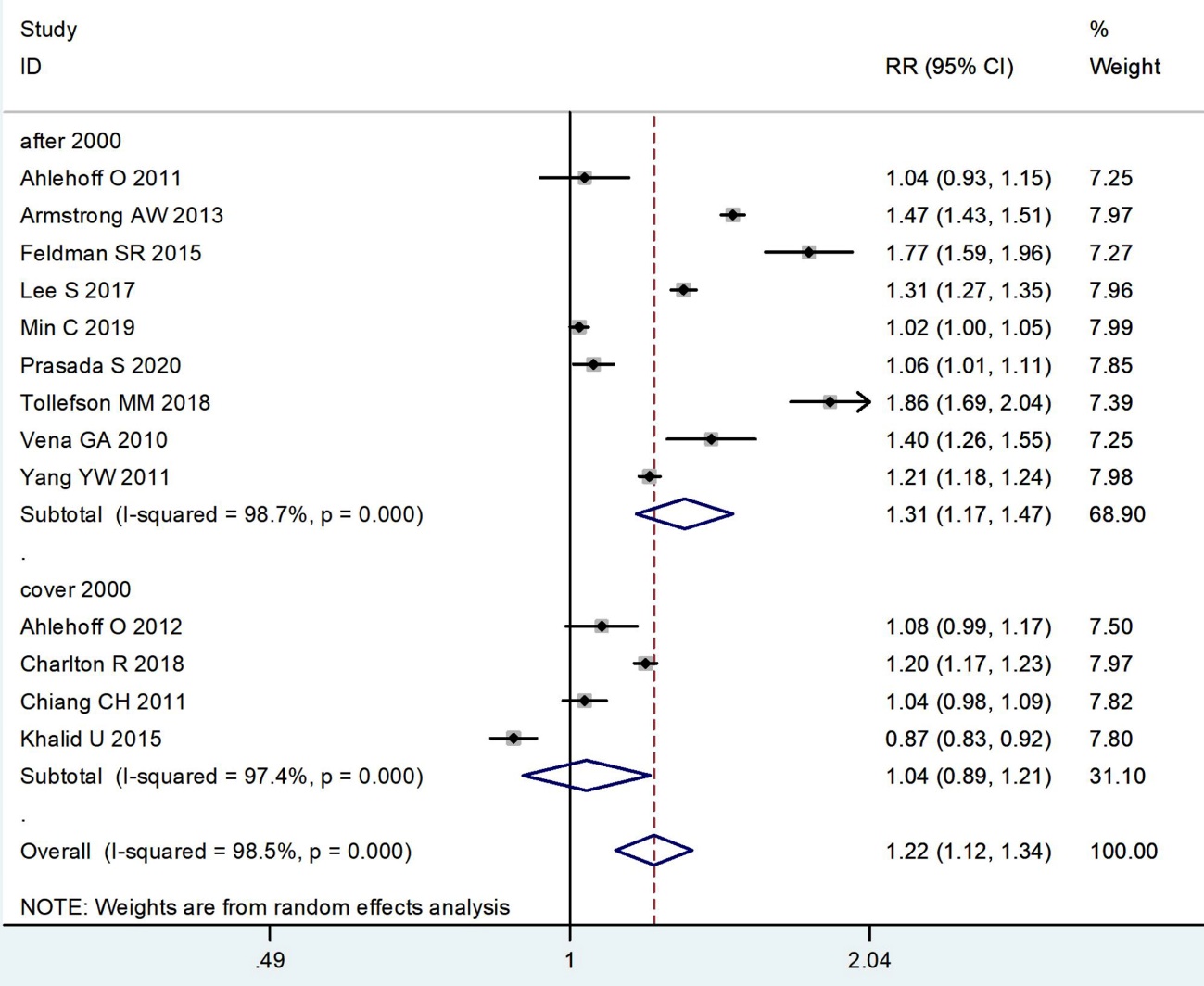


**Supplementary Figure 7** Subgroup analyses in studies with or without funding.


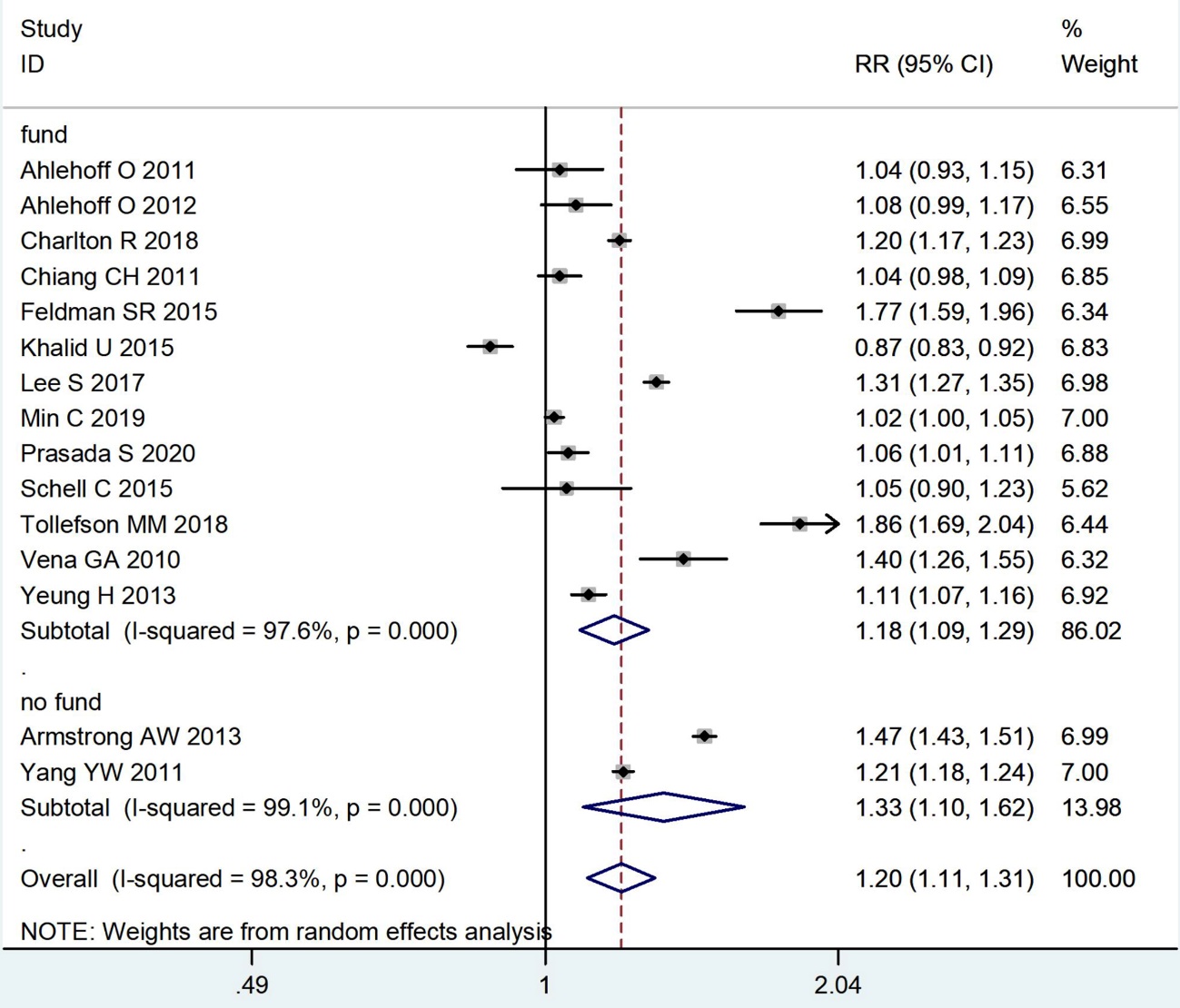


**Supplementary Figure 8** Forest plot of comorbidity risks in psoriatic patients before and after 2006.


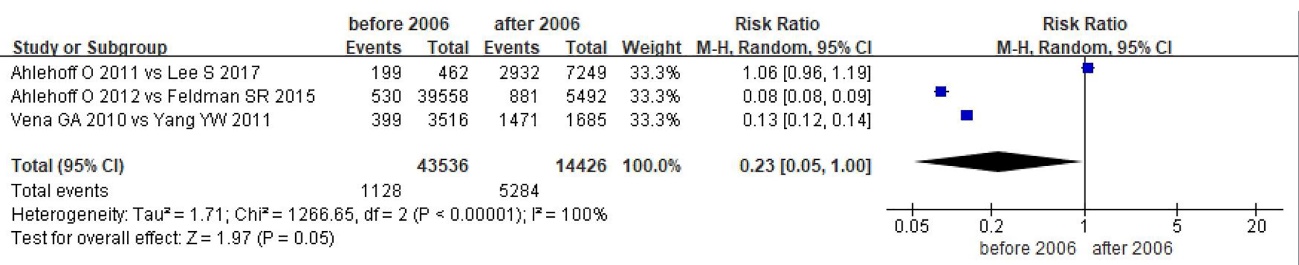


**Supplementary Figure 9** Forest plot of comorbidity risks in psoriatic patients in Asia and West.

**
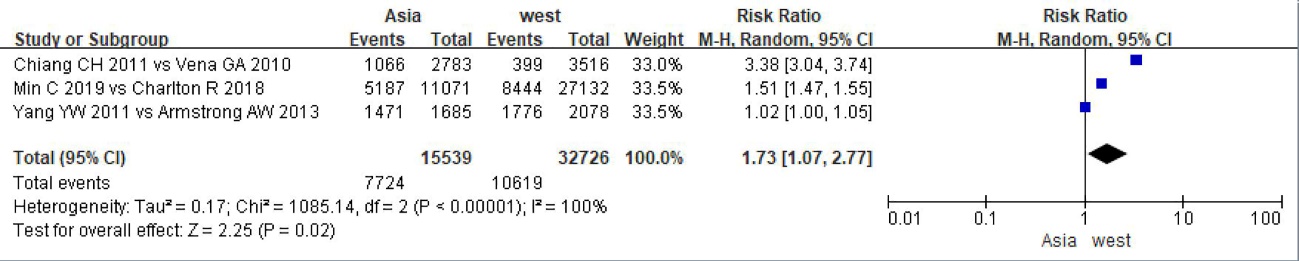
**
